# Unraveling the Role of Natural Sediments in sII Mixed Gas Hydrate Formation: An Experimental Study

**DOI:** 10.3390/molecules28155887

**Published:** 2023-08-04

**Authors:** Mengdi Pan, Judith M. Schicks

**Affiliations:** 1 GFZ German Research Centre for Geosciences, Telegrafenberg, 14473 Potsdam, Germany; mengdi.pan@ucd.ie; 2 School of Chemical and Bioprocess Engineering, University College Dublin, Belfield, D04V1W8 Dublin, Ireland

**Keywords:** mixed gas hydrates, Raman spectroscopy, natural sediments, coexisting phases, hydrate composition, formation kinetics, Qilian Mountain permafrost

## Abstract

Considering the ever-increasing interests in natural gas hydrates, a better and more precise knowledge of how host sediments interact with hydrates and affect the formation process is crucial. Yet less is reported for the effects of sediments on structure II hydrate formation with complex guest compositions. In this study, experimental simulations were performed based on the natural reservoir in Qilian Mountain permafrost in China (QMP) due to its unique properties. Mixed gas hydrates containing CH_4_, C_2_H_6_, C_3_H_8_, and CO_2_ were synthesized with the presence of natural sediments from QMP, with quartz sands, and without sediments under identical p–T conditions. The promoting effects of sediments regardless of the grain size and species were confirmed on hydrate formation kinetics. The ice-to-hydrate conversion rate with quartz sand and natural QMP sediments increased by 23.5% and 32.7%, respectively. The compositions of the initial hydrate phase varied, but the difference became smaller in the resulting hydrate phases, having reached a steady state. Beside the structure II hydrate phase, another coexisting solid phase, neither ice nor structure I hydrate, was observed in the system with QMP sediments, which was inferred as an amorphous hydrate phase. These findings are essential to understand the mixed gas hydrates in QMP and may shed light on other natural hydrate reservoirs with complex gas compositions.

## 1. Introduction

The occurrence of mixed gas hydrates has been proven in Qilian Mountain permafrost (QMP) in China. This deposit is characterized by a shallow depth, namely, 133–396 m below the surface, with a permafrost thickness of only 60–95 m, which is significantly thinner than the Mallik gas hydrate reservoir, with a thickness of about 600 m [1,2,3]. In addition, a more complex gas composition was reported compared to other well-documented permafrost hydrate reservoirs [1,2,4]. The composition and the carbon isotope of the gases dissociated from hydrates indicate that the gas source of this reservoir is composed mainly of deep or crude oil-concomitant gases originating from the coal layers of the Middle Jurassic period, occasionally mixed with a microbial source from shallower depths [5,6,7]. Gas mixtures derived from these deep hydrocarbon reservoirs are being transported from the place of origin through fractures to the shallower hydrate-stability zone for the formation of gas hydrate [8,9].

Gas hydrate samples recovered from QMP contain 60% CH_4_. Besides CH_4_, there is a high percentage of other gas components, like C_2_H_6_, C_3_H_8_, C_4_H_10_, CO_2_, and even heavier hydrocarbons, suggesting the existence of structure II hydrates [1]. Structure II hydrates are characterized by water molecules forming pentagonal dodecahedra (5^12^) and larger hexakaidecahedra (5^12^6^4^) via hydrogen bonds, both stabilized by the inclusion of gas molecules. Raman spectroscopic measurement results indicate that larger hydrocarbon molecules occupy the larger 5^12^6^4^ cavities of structure II hydrates, whereas CH_4_ molecules are mainly in small 5^12^ cavities [10,11]. Higher hydrocarbons may stabilize the larger 5^12^6^4^ cavities better than CH_4_ due to higher guest-to-cavity ratios, which leads to a shift in the equilibrium curve to higher temperatures and lower pressures [12,13]. Therefore, the encasement of hydrocarbons becomes a prerequisite for the formation of the hydrate deposit under these mild pressure and temperature conditions, as observed in QMP. However, the feed gas mixtures are highly dependent on the local conditions. With the presence of a complex gas mixture, the formation of a more-or-less homogeneous hydrate phase is questionable. The coexistence of gas hydrate phases with different structures and compositions may form based on the hypothesis of chemical fractionation of the feed gas phase [14,15,16]. However, laboratory experiments from Pan and Schicks demonstrated that a limited gas supply in the closed system led to an increased enclathration of the available gas molecules with less preference for certain molecules, but is not a prerequisite for the formation of a coexisting hydrate phase [17].

Sediments are unavoidably involved when talking about natural gas hydrates, as the latter are hosted within or beneath permafrost and marine sediments, respectively. It has been shown that the particle size of the host sediments and the mineral composition may affect and alter the hydrate thermodynamic states and the growth kinetics [17,18]. Previous research has indicated an inhibition effect on hydrate growth in fine-grained clayey sediments by synergistic influences of diminished pore water activity in the vicinity of hydrophilic mineral surfaces and the excess internal energy of small crystals confined in pores, which is conceptualized as a “capillary pressure” [18]. However, others have also found that a high concentration of fine grains (<125 μm) led to an explicitly faster gas hydrate formation compared to medium or coarse sands [19]. The relationship between the sediment grain size and hydrate formation seems not straightforward. Certain mineral surfaces (e.g., bentonite) can promote heterogeneous nucleation of hydrate [20]. Some organic substrates, such as kerogens or biofilms, may also possibly promote hydrate precipitation [18]. In the drilling cores from QMP, the major strata encountered include the Middle Jurassic lacustrine coal-bearing Jiangcang formation and below it the Muli formation. The Jiangcang formation is mainly composed of black and gray oily shale and mudstone, mixed with gray siltstone and fine-grained sandstone; the Muli formation consists mainly of gray whitish siltstone and fine-grain sandstone [21]. Less is known about the potential effects of these sediments and mineral compositions on the mixed gas hydrate formation kinetics and the thermodynamic properties of the resulting hydrate phase. Therefore, we investigated this effect and compared the results with the effect of quartz sand and the hydrate formation process without sediments. The results are presented in this paper, as we think that these findings are of importance to better understand the mixed gas hydrate system in QMP.

## 2. Results and Discussion

### 2.1. Influence of Sediments on the Formation Kinetics of Mixed Gas Hydrates

In the closed laboratory simulation system (batch pressure vessels), pressure drop was used as an indicator for the formation of gas hydrates on a mass-balance basis, as it reflected the enclathration of gas molecules into the hydrate structures, leading to a decrease in the absolute pressure in the system.

**Figure 1:**
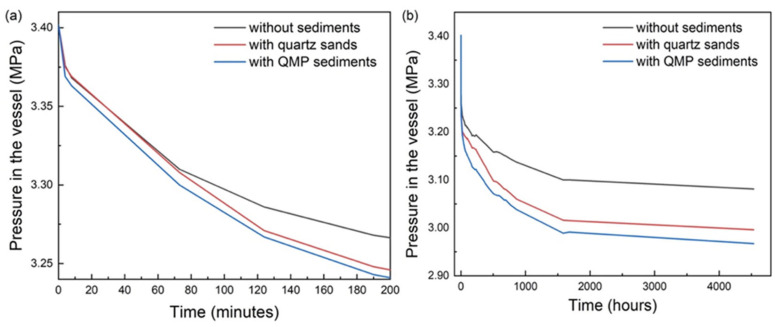
Pressure–time diagram of three parallel tests, representing the experiments without sediments (black curve), with quartz sands (red curve), and with QMP sediments (blue curve) during the first 200 min (**a**) and for the entire experimental period (**b**).

Mixed gas hydrates were formed in batch pressure vessels at 268 K and 3.4 MPa and thus at p–T conditions far within the stability field based on the thermodynamic model calculation (CSMGem) [22]. Figure 1 provides the absolute pressure in three vessels over time. The curves are shown at two different time scales, where (a) is during the first 200 min and (b) is throughout the entire experiment, which lasted for 4500 h. Figure 1a clearly shows that in all three tests there was a sharp drop in pressure right at the beginning of the tests, indicating a quick formation of mixed gas hydrates when the ice powder was first exposed to the gas mixture. Among them, the fall in the pressure in the system containing QMP sediments was the most significant in the first 10 min. However, the pressure curve with quartz sand almost overlapped the curve without sediments within the first 70 min. The formation of hydrates in the vessel with quartz sand accelerated and a more pronounced pressure decrease was observed compared to the group without sediments. This observation is in agreement with the phenomenon observed by Heeschen et al. that the presence of sand particles with a grain size > 500 µm does not necessarily result in a faster hydrate formation within the first 48 h [23]. Thereafter, the formation process continued but the formation rates were considerably slower in all three tests, as the slope of the curves gradually flattened after around 1500 h (Figure 1b). This might be explained as the diffusion-controlled process during the continuous transformation of ice to hydrates after a hydrate layer had formed around the ice particle surface at the very beginning of the experiment. The system with QMP sediments showed the highest total gas consumption until the end of the experiments. Despite a somewhat similar beginning of hydrate formation in the other two systems, the presence of quartz sand still displayed promoting effects since the final absolute pressure was lower than that in the system without sediments, indicating that more ice powder had transferred to mixed gas hydrates. After the repetition of the above experiment on a time scale of 168 h, a similar phenomenon for the hydrate formation kinetics was observed.

Apparently, the presence of sediments, regardless of the grain size and species, was favorable for gas hydrate formation. A statistical calculation, based on the pressure drop and the volume of the reactor, revealed that in vessel No. 1 without sediments, a total of around 7.7 mL mixed gas hydrates formed during the experimental period, whereas the amount of hydrates was 9.5 mL in vessel No. 2 with quartz sands and 10.2 mL in vessel No. 3 with QMP sediments. Since 34 mL of ice powder were originally provided in all three systems, the ice-to-hydrate conversion rate in these three vessels reached 22.6%, 27.9%, and 30.0%, respectively. With the presence of sediments, the conversation rate increased by 23.5% for quartz sand and 32.7% for natural samples. There is no doubt that the difference in the hydrate formation rate and the amount in the three batch vessels was governed by the sediments and their matrix characteristics.

**Figure 2:**
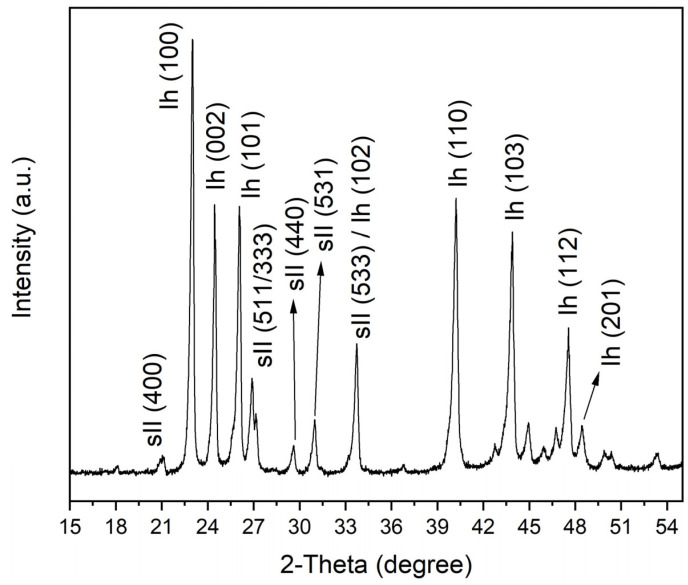
Powder X-ray diffraction patterns of hydrate samples formed in the pressure vessel with sediment powder from QMP.

The powder X-ray diffraction analysis of the recovered samples in the three vessels confirmed the occurrence of structure II hydrates. Figure 2 depicts the XRD patterns taken from the samples recovered from vessel No. 3 with QMP sediments. The results confirm that even in vessel No. 3, which had the highest pressure drop, only a limited amount of the ice powder converted to mixed gas hydrates within the 4500 h.

A preferential occurrence of gas hydrate formation in coarse sandy sediments was previously confirmed for both field observations [24,25] and laboratory experiments [26,27,28]. Clennell et al. [18] ascribed this observation in his conceptual model to higher driving forces for gas hydrate formation in larger pores based on a smaller influence of capillary pressure and a smaller depression of water activity, both caused by fine-grained particles. However, the observation of a faster hydrate formation in fine-grained QMP sediments in this study at the very beginning of the experiment is in good agreement with the results from experimental simulations of Heeschen et al. [23], who reported a shorter induction time for the methane hydrate formation in the <125 µm sediment group compared to other coarser sediment groups. A closer look at the pressure curve during the further course of the experiment (>120 min) in Figure 1 reveals that the slope of the curve was about the same, i.e., the further conversion of ice to hydrate occurred at a similar rate in the two sediment-containing samples.

It is generally accepted that the guest molecules are first dissolved into the aqueous phase before they are encased in hydrate cavities. However, in the case of hydrate formation from ice, a liquid water phase is not a prerequisite for hydrate formation, because a quasi-liquid layer may exist between the forming hydrate layer and the surface of the unreacted ice particle [29]. This layer is believed to be a thin mobile phase of water molecules with mobilities that are intermediate between those of liquid water and crystalline ice [30]. For hydrate formation, this thin layer of mobile water molecules needs to be supersaturated. With the presence of sediments, a third interface allowed for a reduction in the nucleation work [20]. A smaller grain size range had larger surface area; thus, the heterogeneous nucleation opportunities were enhanced, resulting in an increase in the hydrate formation rate [31]. In addition, the occurrence of mineral particles with a small grain size may enhance the absorption of gas molecules to its surfaces, which leads to an earlier local super-saturation.

QMP sediments showed a larger compositional difference, as indicated by the XRD results. Minerals other than quartz accounted for more than 20% of the sediments in this sample, mainly including feldspar, mica, and kaolinite. In contrast, the sand samples consisted of 99% quartz. As stated by Blackwell in her PhD thesis [32], specific particles, such as CuO, αAl_2_O_3_, γAl_2_O_3_, and CaCO_3_, act as effective gas hydrate nucleators, which are also among the best ice nucleators and exhibit small crystal lattice mismatches with ice (hexagonal). In a similar way, the occurrences of K-feldspar, mica, and kaolinite in QMP sediments could possibly offer preferential conditions for hydrate nucleation. Adsorption of water at the enlarged surface of hydrophilic aluminosilicate may lead to a reduced free water phase having to be saturated with gas molecules.

The potential influence of salts, which are known to be inhibitors to gas hydrate formation [22], remained uncertain in this experiment, as the initial salinity for these two sediments was not determined.

### 2.2. Effect of the Sediments on the Thermodynamic Properties of Mixed Gas Hydrates

It should be mentioned that the potential changes in gas composition in the ordered gas mixture during storage and transportation requires the determination of the exact composition of the gas phase before each experiment. Table 1 summarizes the information of the ordered gas mixture as well as the actual gas mixture before six repeated tests. Obviously, the composition of the gas mixture that flew into the optical pressure cell differed from the order composition, containing a lower concentration of CH_4_ but higher concentrations of other hydrocarbons and CO_2_. The last two tests with quartz sand were carried out with gas mixtures containing even lower concentrations of CH_4_. This might have been caused by the molecular weight difference in the gas mixture, as CH_4_ was the lightest molecule and might have been released faster when the gas cylinder was first opened. Accordingly, further discussion and calculations of the hydrate composition at equilibrium in CSMGem (Table 1) referred to the measured gas composition rather than the ordered gas composition.

**Figure 3:**
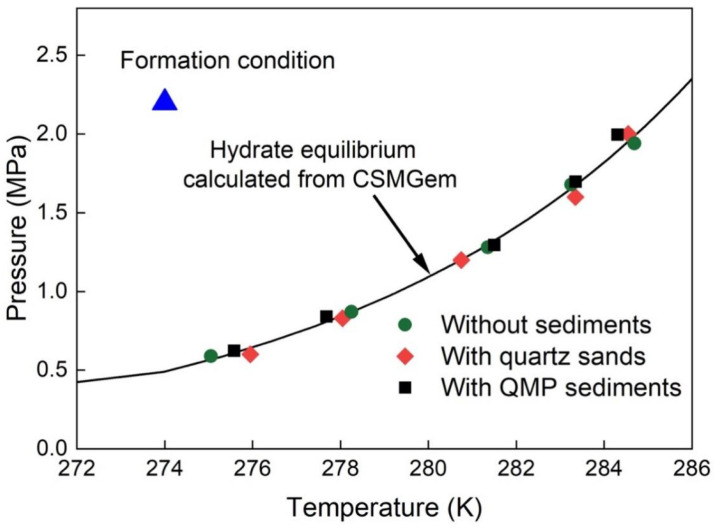
Phase diagram of mixed gas hydrates. Experimental data are plotted as data points. The black line represents the modeled data obtained from CSMGem [22]. The blue triangle stands for the chosen p–T condition for the formation of the mixed gas hydrate, which falls within the stability field.

Natural gas hydrates generally occupy pores in the sediment matrix, and hence, the pore size distribution influences the thermodynamic equilibrium curve of the gas hydrates [26,33,34]. Experiments imply that the equilibrium temperature decreased with the decrease in pore diameter at a given pressure condition (see Malagar et al. [35] and references therein). Figure 3 presents the p–T diagrams for the studied mixed gas hydrates. The formation condition (blue triangle) of the time-dependent experiments was well within the stability field. All phase data in three separate systems were experimentally determined (displayed as data points). It can be concluded from the figure that the experimental data in all three different systems corresponded well with the modelled data (black curve) acquired from CSMGem for mixed gas hydrates formed without sediments. In other words, the presence of sediments in the aqueous phase did not affect the hydrate equilibrium conditions in this study.

It should be noted that only 0.02 g of sediments were added to the pressure cell together with 150 µL of deionized water. A rough estimate shows that the amount of sediments represented about 13 wt% of the dispersion in the system. Hence, sediment particles, whether fine grains or coarse sands, were limited in the high-pressure cell. A recent study from Bhawangirkar et al. [36] investigated the thermodynamics of methane hydrate formation and dissociation in the presence of sediments from a natural reservoir at varying concentrations in an aqueous solution. It was concluded from the experiment that natural sediments have an inhibition effect on the hydrate equilibrium, with the equilibrium being shifted towards lower temperatures and higher pressures. Nevertheless, the inhibition effect was also invisible with 10 wt% sediments in solution, which depicted a similar equilibrium curve as that of pure methane hydrates without sediments. We therefore hypothesize that the amount of sediments in our experiments was also too small to observe a measurable influence.

### 2.3. Effect of the Sediments on the Composition of Mixed Gas Hydrates

**Figure 4:**
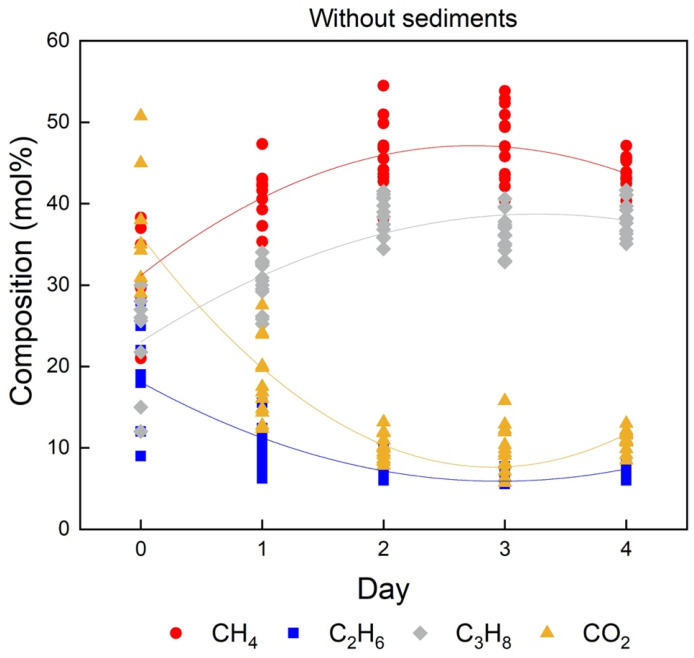
Changes in the hydrate composition throughout the whole experimental period, which were measured in one of the first two tests without sediments. The data points in varied colors indicate the concentrations of different guest components in the hydrate phase measured from the selected hydrate crystals. The solid curves of corresponding color show the average concentration changes of the respective component in the hydrate phase, which are fitted by smooth curves.

During the hydrate formation process in the in situ experiments, the p–T conditions were kept constant, and the gas flow was supplied continuously. This was done to avoid changes in the gas phase due to a preferred enclathration of some components in the hydrate phase. Raman spectra recorded changes of hydrate compositions for 15 selected crystals over the formation period until the system reached a steady state. The first day of hydrate formation was designated as Day 0. In the first two tests without sediments (Figure 4), it was found that C_3_H_8_ and CO_2_ molecules were preferentially enriched in the hydrate phase from the beginning of formation compared to their corresponding concentrations in the gas phase (Table 1). However, the concentration of C_3_H_8_ in the hydrate phase continued to increase as the experiment proceeded, whereas the content of CO_2_ in the hydrate phase decreased with time. It also turns out that the concentration of CH_4_ in the hydrate phase increased during the experiment, following a similar trend as C_3_H_8_. As indicated by Pan and Schicks [17], C_2_H_6_ shows a relatively higher solubility in the water phase compared to CH_4_ and C_3_H_8_ but has less pronounced ability to stabilize the larger 5^12^6^4^ hydrate cavities compared to C_3_H_8_. This might be the likely reason for the observation of a decrease in C_2_H_6_ content in the hydrate phase during the experiment. Moreover, large variations in the hydrate compositions among the analyzed crystals were observed at the beginning of the experiment. With the progression of the experiment, the variation became smaller and the system reached a steady state, as indicated by the data points in this figure. The average composition of the resulting hydrate phase at the end of the experiment contained around 43.6 ± 2.34 mol% CH_4_, 7.0 ± 0.51 mol% C_2_H_6_, 38.6 ± 2.21 mol% C_3_H_8_, and 10.8 ± 1.21 mol% CO_2_.

**Figure 5:**
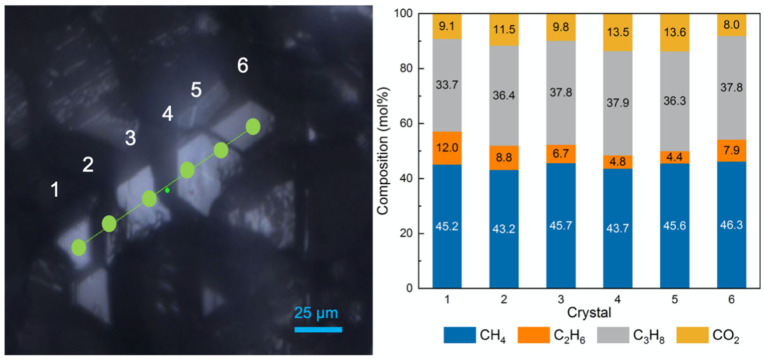
(**left**) Raman line scans across the surface of five hydrate crystals, (**right**) composition variations in the hydrate phase at 6 measuring points across the 5 hydrate crystals.

For a better understanding of the spatial distribution of gas molecules in the hydrate phases, a total of five well-structured crystals located in a straight line were selected in the first test without sediments for line mapping. Altogether, six measuring points were chosen on the surface of the selected crystals, as shown in Figure 5. It should be pointed out that the fourth and fifth measuring points were on the surface of the same hydrate crystal. These points were scanned step by step automatically in one direction. The results, presented as stacked columns, show pronounced variations in the composition of the hydrate phase for six measurement points. It should be noted that the accuracy of Raman measurements was tested before the experiments and showed excellent reproducibility [37]. Therefore, the measured variations in local composition of the hydrate crystals (Figure 5) demonstrated the non-stoichiometric and heterogeneous character of the hydrate phase formed from a complex gas mixture, even though pronounced fluctuations in the composition of the gas phase during hydrate formation could be ruled out due to the continuous gas flow.

**Figure 6:**
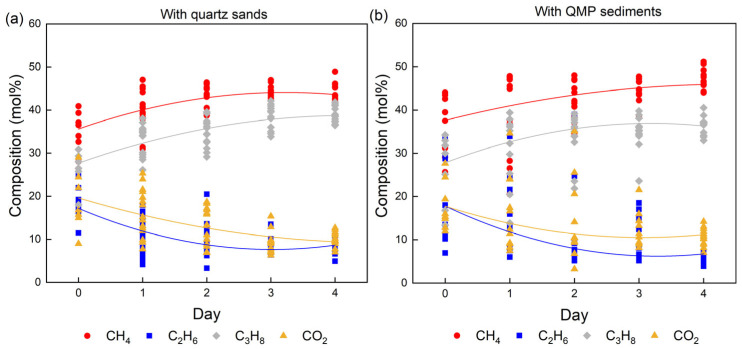
Changes in the hydrate composition throughout the whole experimental period, which was measured in systems with quartz sands (**a**) and QMP sediments (**b**). The data points in varied colors indicate the concentrations of different guest components in the hydrate phase measured from the selected hydrate crystals. The solid curves of corresponding color show the average concentration changes of the respective component in the hydrate phase, which are fitted by smooth curves.

With the occurrences of sediments, whether quartz sand or QMP sediments, the hydrate compositions were totally different at the beginning of the formation process compared to the hydrate composition without the presence of sediments (Figure 6 and Table 2). On Day 0, the concentration of CO_2_ in the gas hydrate phase formed without sediments (Figure 4) was considerably high, accounting for more than 35 mol% on average. Interestingly, the amount of CO_2_ enclathrated in the hydrate phase in the presence of sediments (Figure 6a,b) barely reached 30 mol%. Meanwhile, CH_4_ and C_3_H_8_ reached relatively high concentrations on Day 0, as shown in Figure 6a,b. This may indicate that the development of the hydrate systems towards steady states varied in the three systems. In other words, specific guest molecules were encased in the hydrate cavities more rapidly in the presence of sediments compared to those without sediments. Therefore, in the system without sediments, a higher concentration of CO_2_ was already detected in the hydrate phase at the beginning of the experiment. The experiments with quartz sands and QMP sediments reflected similar trends as the previous background test for the changes in composition in the hydrate phase over time: The concentrations of CH_4_ and C_3_H_8_ increased with time, whereas the amount of C_2_H_6_ and CO_2_ molecules decreased. Noteworthy, the decrease in relative C_2_H_6_ concentration in the resulting hydrate phase was quite prominent with the QMP sediments (Table 2). Finally, the formed hydrate composition in all three systems did not show a great difference, leading to the conclusion that the presence of sediments played a minor role in affecting the composition of the resulting hydrate phase at a steady state.

As can be seen from Figure 6b and Table 2, the variation in the hydrate composition in the presence of QMP sediments was quite high during the first two days, which was also observed in the other two tests without sediments and with quartz sand. In the following days, however, the variations in the hydrate composition became smaller when quartz sand or no sediments were present. This was not the case with QMP sediments. It was observed that on Day 2 and Day 3, the compositions of a few hydrate crystals were significantly different than the others.

### 2.4. Coexisting Phases Formed with the Presence of QMP Sediments

It was demonstrated from PXRD measurements (Figure 2) that only structure II hydrates were observed in the system with QMP sediments. However, the data points collected on Day 2 and Day 3 using Raman spectroscopy for the analysis of the hydrate phase in the system with QMP sediments (Figure 6b) were far from the average composition line, probably indicating the coexistence of another phase with a different composition.

**Figure 7:**
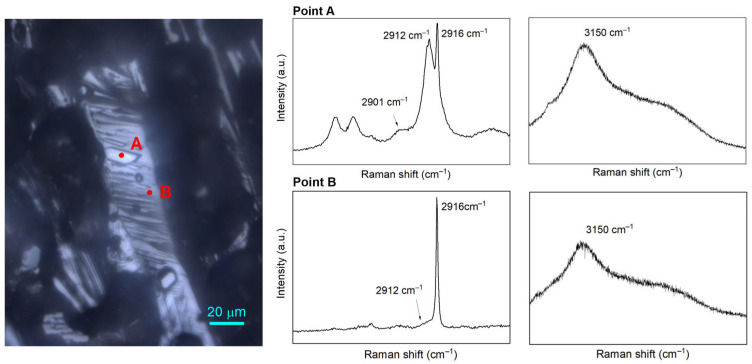
Microscopic observation of two selected points: point A and point B (**left**); and the corresponding Raman spectra of the two points for C–H stretching vibrational modes ranging from 2850 to 2950 cm^−1^ and O-H stretching vibrational region ranging from 3000 to 3600 cm^−1^ (**right**).

Analysis of the Raman spectra for two measuring points on Day 3 in the system with QMP sediments yielded the expected results (Figure 7). Point A in Figure 7 exhibited a well-developed hydrate crystal with sharp edges, whereas point B seemed to measure the flat base located below the hydrate crystals. It is known that the ratio of large-to-small cavities for structure I and structure II hydrates is 3:1 and 1:2, respectively. The integrated Raman band intensities of the C–H vibrational modes should be good indicators of the hydrate structure. The assigned Raman bands are summarized in Table 3. It turns out that Raman bands measured on the surface of point A indicated the presence of typical structure II hydrates, with CH_4_ occupying mainly the small 5^12^ cavities of structure II but also a small proportion in the large 5^12^6^4^ cavities. CO_2_, C_2_H_6_, and C_3_H_8_ were incorporated into the large 5^12^6^4^ cavities of the structure II hydrates. Nevertheless, Raman spectra taken at point B did not exhibit the typical structure I or structure II hydrate signal. Instead, weak Raman bands and a very strong CH_4_ gas peak were observed, indicating a metastable solid phase with only a limited amount guest molecules incorporated into the hydrate cavities. In the O–H stretching region, a prominent broad peak at 3150 cm^−1^ followed by a weak shoulder was observed at point A, whereas this band was less pronounced at point B, leading to the speculation of a coexisting solid phase with fewer hydrogen-bonded water molecules existing at point B than at point A [38].

**Figure 8:**
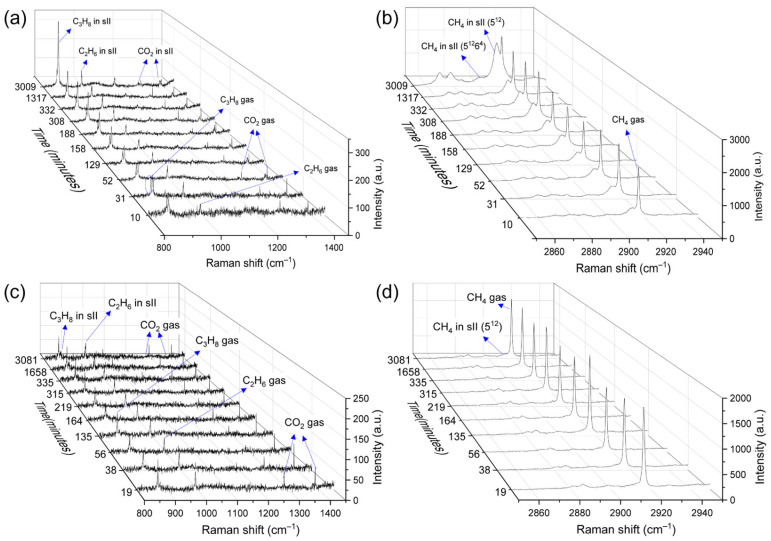
Real-time Raman spectra monitoring the two different formation patterns measured from point A (**a**,**b**) and point B (**c**,**d**). (**a**,**c**) C–C stretching vibrational modes ranged from 800 to 1450 cm^−1^. (**b**,**d**) C–H stretching vibrational modes ranged from 2850 to 2950 cm^−1^.

By continuous characterization on measuring points A and B (Figure 7), time-resolved Raman spectra were acquired during the hydrate formation process. In Figure 8a, b, which refers to the changes in point A, a small Raman band at 2912 cm^−1^ was observed beside the gas peaks after 10 min. This could be assigned to CH_4_ molecules in the small 5^12^ cavities of structure II hydrates. In the meantime, Raman bands at 1274 cm^−1^ and 1381 cm^−1^ for CO_2_ in the hydrate phase, together with the peak at 876 cm^−1^ for C_3_H_8_ in large 5^12^6^4^ cavities of structure II hydrates, were also detected in the Raman spectra. Thereafter, a Raman band arose at approximately 991 cm^−1^, corresponding well with the literature data for C_2_H_6_ molecules in the 5^12^6^4^ cavities. The intensities of these above-mentioned Raman bands increased gradually as the experiment proceeded, showing typical characteristics of structure II hydrates. Regarding the Raman measurements taken at point B, the Raman spectra showed a poor signal-to-noise ratio (Figure 8c,d). Only gas peaks at 2916 cm^−1^, 869 cm^−1^, 993 cm^−1^, 1285 cm^−1^, and 1387 cm^−1^ were present during the first few minutes. Later on, CH_4_ was shown to be encased in the small 5^12^ cavities of hydrate crystals as a shoulder emerged next to the CH_4_ gas peak at around 2912 cm^−1^. The enclathration of other hydrocarbons and CO_2_ molecules in the hydrate phase was indicated by various tiny peaks (Figure 8c), suggesting an extremely slow process. Continuous microscopic observations of the hydrate phase revealed that with the expansion of the hydrate crystals, the metastable solid phase was covered by a layer of the well-developed crystals. Therefore, it was not possible to track the same measuring point B on the last day. This might explain the small variation in the hydrate crystals measured on Day 4, as shown in Figure 6.

Based on the experimental condition (274 K) and the obtained Raman spectra, the existence of ice or structure I hydrates as a coexisting solid phase could be ruled out. It is likely that the metastable solid phase corresponds to an amorphous hydrate phase, which was previously reported by Jacobson et al. through molecular dynamics simulations [41]. They described the nucleation of hydrates as a multistep mechanism and proposed the formation of so-called “blobs” that consist of gas molecules separated by half-cages of water molecules in the aqueous solution. When the “blobs” reach a critical size, water molecules agglomerate and are locally ordered, forming the amorphous hydrate phase. The tiny Raman bands for CH_4_ and other hydrocarbons observed in the samples with QMP sediments (Figure 8c,d) may indicate the formation of amorphous hydrate phases with partial enclathration of guest molecules. Thereafter, the amorphous hydrate nucleus rearranged and formed crystalline structure II hydrates at the end of the experiment. Interestingly, a similar phenomenon was also observed in the structure I CH_4_ hydrate system, which displayed a metastable structure II hydrate as a coexisting pre-hydrate phase apart from the well-structured methane hydrate crystals [42].

It is worth mentioning that the occurrence of the coexisting amorphous phase apart from the initial structure II hydrates was not observed without sediments or with quartz sands. It was therefore assumed that QMP sediments with unique physical and chemical properties led to the formation of the metastable phase during the formation process. As can be seen in the Materials and Methods section, QMP sediments have oily features, which makes it difficult for them to be dispersed into the water phase. Even after careful stirring, a thin layer floated on top of the water surface, suggesting the existence of specific minerals or organic compounds like fats, oils, or petroleum products that were hydrophobic. At specific points around the hydrophobic minerals or organic compounds, fewer water molecules were available for further agglomeration. Inspired by hydrophobic amino acids as novel kinetic inhibitors for gas hydrate formation [43], the hydrophobic particles in some specific areas might also have perturbed the arrangement of water molecules. Hence, the nucleation and growth of hydrates in these areas might have been retarded or even prevented. Consequently, Raman spectra of point B somehow exhibited an amorphous hydrate characteristic but not a crystalline phase.

## 3. Materials and Methods

To understand the complex mixed gas hydrate system in QMP, an experimental simulation was carried out to investigate the effects of local sediments on the mixed hydrate formation process. In this study, a gas mixture containing CH_4_, C_2_H_6_, C_3_H_8_, and CO_2_ was used for the synthesis of mixed gas hydrates. Two parallel experimental series were carried out with two kinds of sediments separately: natural samples from QMP and quartz sand. A background test was performed without sediments under identical p–T conditions. The natural sample was retrieved from a branch well SK-2 and at a depth of 355 m (5 m below the hydrate enrichment layer) in the field test production of gas hydrates in 2016 in the Muli area, QMP [44,45]. In situ Raman spectroscopic measurements and microscopic observation were employed for the investigation of the hydrate formation process on a μm scale and to determine the composition of the forming hydrate phase as well as the coexisting gas phase. Through continuous characterization of the gas and hydrate phases during the process, important information about the gas enclathration with/without the presence of sediments was revealed. As a supplement, the same mixed gas hydrates were also synthesized in three medium-volume pressure vessels with/without sediments for the exploration of formation kinetics (ex situ measurements).

**Figure 9:**
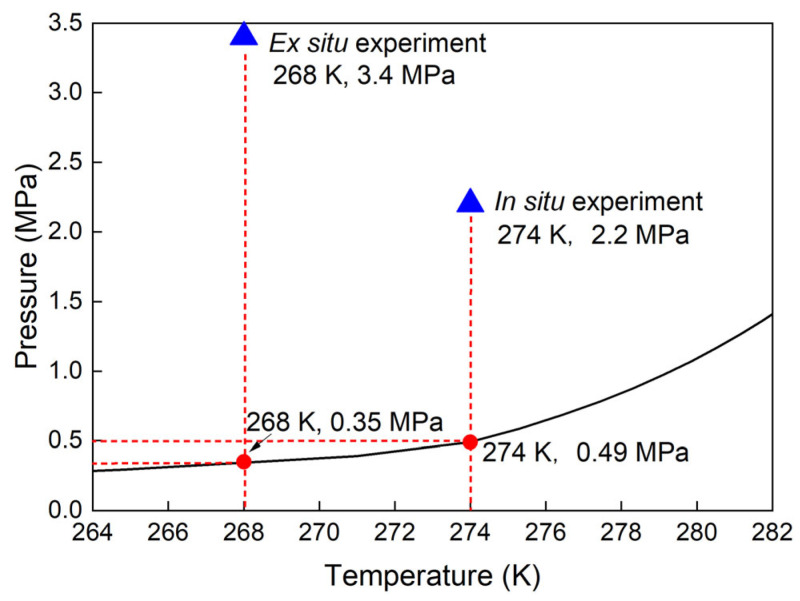
Experimental conditions applied for in situ and ex situ experiments in this study. The curve indicates the equilibrium p–T conditions for the mixed gas hydrates calculated from the software CSMGem [22].

In situ: Mixed gas hydrates were initially synthesized in an optical pressure cell with a volume of 550 µL from pure water and a gas mixture at 2.2 MPa and 274 K (Figure 9). As indicated above, the gas composition detected from the dissociation of natural hydrates recovered in QMP contains a mixture of hydrocarbons and CO_2_ in addition to CH_4_. Therefore, a gas mixture with a defined composition of 63 mol% CH_4_, 15 mol% C_2_H_6_, 15 mol% C_3_H_8_, and 7 mol% CO_2_ (provided and certified by Rießner–Gase GmbH, Lichtenfels, Germany) was used in this study. Since the defined composition (±0.1 mol% rel) of the gas mixture is only guaranteed by the manufacturer for storage at certain temperatures and for a limited period of time, Raman spectroscopic measurements were carried out on the gas phase before each test and twice a day during the test to determine the actual relative composition (in mol%).

**Figure 10:**
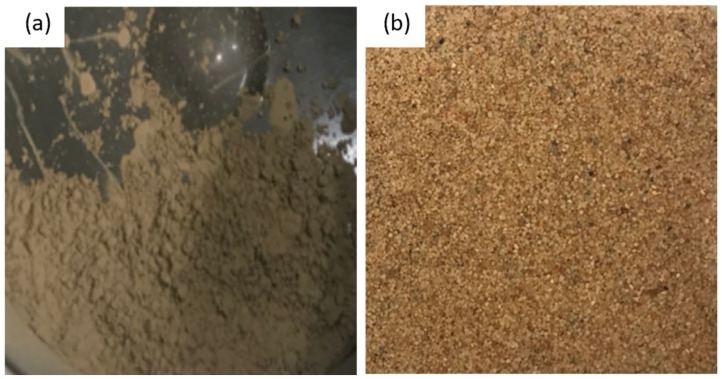
Photo of the sediment samples: (**a**) powder milled from the drilling core sample retrieved in QMP, (**b**) quartz sand particles.

Initially, a natural core sample retrieved from QMP was ground into powder with a grain size of <63 µm, confirmed by sieving. Powder X-ray diffraction (PXRD) analysis was performed using a PANanalytical Empyrean powder diffractometer with a PIXcel^3D^ detector, automatic divergence, and antiscatter slits with CuKα radiation. The mineral phases were identified using the program EVA (by Bruker) and quantified with the open-source program Profex. Detailed information about the PXRD measurements can be found in our previous publication [46]. It turns out that the core sample was mainly composed of 76.5 wt% quartz, 9 wt% plagioclase, 1 wt% K-feldspar, 6.5 wt% mica, 5 wt% kaolinite, and 2 wt% ankerite/siderite [46]. The pore size of the original drilling core sample ranged from 0.1 to 10 µm, and the particle sizes ranged from 0.01 to 30 µm. The highest relative pore volume was detected at 0.7 µm, whereas the particle size of 1 µm obtained the highest relative percentage of sample volume [46]. Considering the fact that sandy sediment usually holds a higher saturation of gas hydrates in natural occurrences [47], quartz sand provided by Schlingmeier Quarzsand GmbH & Co. KG (Gross Schwülper, Germany) was also applied in this experiment. The particle grain size of the quartz sand (~99.6 wt% SiO_2_) was between 250 µm and 1000 µm. Figure 10 depicts the photo of two sediment samples, with (a) the powdered sediments from QMP and (b) the quartz sand.

**Figure 11:**
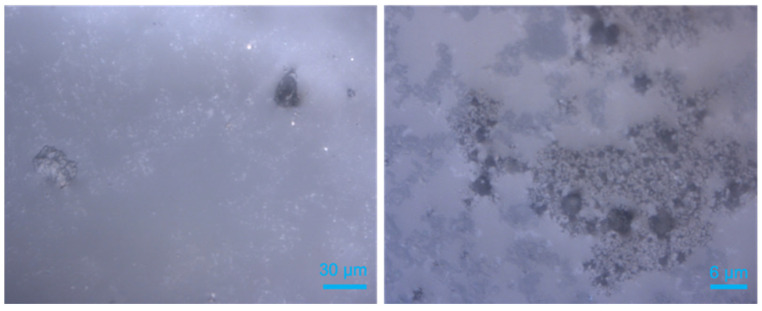
Microscopic observations of sediments in aqueous phase before pressurization: (**left**) quartz sand, (**right**) QMP sediments.

During the experiments for in situ Raman measurements, 0.02 g of quartz sand were directly placed into the optical pressure cell before adding 150 μL deionized water. It should be mentioned that sediment powder from QMP exhibited oily features; instead of being easily dispersed in water, they tended to float on the water surface. In this case, a sediment load of 0.02 g was first mixed with 150 μL of deionized water. The mixture of sediments and deionized water was carefully stirred to ensure a good mixing before loading into the pressure cell. Thereafter, the cell was sealed and pressurized with the above-mentioned gas mixture at 2.2 MPa. It should be noted that neither the QMP sediments nor the quartz sand were compacted but presented as loose sediments in the aqueous phase. Thus, the sediment grains were not present in a specific arrangement with a defined pore volume. The optical cell experienced an alternate cooling–heating–cooling process before it reached the final temperature of 274 K. The p–T condition was chosen to avoid the formation of ice during the growth of euhedral hydrate crystals. Figure 11 shows the microscopic pictures of the water-saturated quartz sand (left) and powdered QMP sediments in water (right).

The sample cell was supplied with a continuous gas flow to avoid any changes in the feed gas composition during the hydrate formation process. Single-point Raman spectroscopic measurements were continuously carried out in the gas phase and on the hydrate crystals using a LabRAM HR Evolution dispersive Raman spectrometer from HORIBA Jobin Yvon GmbH (Oberursel, Germany) with 1800 grooves/mm grating. The optical cell was integrated onto a motorized, software-controlled, Märzhauser Scan + sample stage attached to a microscope with a 20× objective (Olympus, Hamburg, Germany). The excitation source for the Raman spectrometer was a frequency-doubled Nd:YAG solid-state laser with an output power of 100 mW working at 532 nm. The Raman spectrum of neon light (full width at half maximum at 1706 cm^−1^) was used for the determination of the spectral resolution, which was 0.5 cm^−1^ under a selected focal length of 800 mm. By using the 20× objective and selecting a pinhole number of 30 μm, an optimum spatial resolution of 1.6 μm in the planar direction and 6.2 μm in the *z* direction was reached. The silicon peak (521 cm^−1^) was employed for the calibration of the Raman band positions. For a better understanding of the spatial distribution of the hydrate phases, well-structured crystals were selected for hyperspectral mapping.

The measurements continued for 5 days until the systems reached a steady state in which no further changes in hydrate composition or morphology were observed. Each parallel test was repeated two times. The molar composition of the gas phase and hydrate phase was calculated semi-quantitatively based on the method described by Beeskwo-Strauch et al. [48]. More details regarding the optical pressure cell, the Raman system, and the data analysis methods can be found elsewhere [37].

For the determination of the hydrate equilibrium condition, various pressure conditions were applied. After the initial formation of mixed gas hydrates at a specific p–T condition, the temperature of the system was gradually increased (0.5 K/min) to melt most of the hydrate crystals, leaving only residual structures. Thereafter, the system was cooled down at a relatively slow rate (1 K/10 min). The temperature point was designated as the equilibrium temperature under the selected pressure condition when hydrate recrystallization was first observed at that moment in the system. The same procedures were repeated under 5 varied pressures to investigate the phase diagram of mixed gas hydrates.

Ex situ: For the determination of the influence of sediments on the hydrate formation kinetics, mixed gas hydrates were synthesized from ice powder and the same gas mixture in three batch pressure vessels featuring a 420 mL inner volume in parallel. To generate ice powder, deionized water was first subjected to a liquid nitrogen bath and powdered in a Spex 6750 Freezer Mill that had been cooled with liquid nitrogen. The ice particles were crushed by a pestle driven by a cryo-magnet at 77 K for a duration of 120 s. By using ice powder instead of water, the system achieves a larger surface area and a more homogenous distribution of the water molecules in the sediment, which promotes the hydrate formation process.

The batch pressure vessels were pre-cooled to 268 K to minimize the melting of ice and to avoid thermal gradient. Vessel No. 1 was solely filled with 34 mL ice powder. Vessel No. 2 was filled with 34 mL ice powder combined with 8 g sediment powder from QMP, whereas vessel No. 3 had the same amount of ice powder but with the addition of 8 g quartz sand. The selected amount of ice was precisely determined based on the calculated composition of the hydrate phase at equilibrium that would be formed with the chosen gas mixture using CSMGem. We calculated the maximum amount of ice (34 mL) that could reach the equilibrium composition at complete conversion if the pressure vessel was pressurized with 3.4 MPa gas mixture. We are aware that this approach does not prevent the change in the gas phase due to the preferential inclusion of specific components in the hydrate phase, but it ensures that all components are retained in the gas phase to a sufficient extent.

Afterward sediment and ice were added to the three pressure vessels, they were sealed and pressurized with the same gas mixture at around 3.4 MPa before being stored in a freezer at 268 K (Figure 9). The pressure drop in each vessel was continuously monitored throughout the formation process. A fast pressure decrease at the beginning of the process indicated the formation of mixed gas hydrates. Thereafter, the pressure decrease slowed down and gradually flattened out as the reaction proceeded. After 4500 h, the system reached a steady state in which no pressure changes were observed over 3 days. The amount of formed gas hydrates and the ice–hydrate conversion rate could be estimated from the recorded pressure drop in each vessel. The formation kinetics were reflected by the rate of pressure decrease over time.

To conduct the ex situ X-ray diffraction measurements, the recovered sample materials from the batch vessels comprising hydrates and ice were quickly quenched in liquid nitrogen immediately after depressurization. All preparation work was conducted in a cooled glove box at 268 K to minimize hydrate dissociation and the risk of air moisture condensing onto the samples. Similar to the above-mentioned ice particles, the samples were also ground into powder in the freezer mill (Spex CertiPrep, Metuchen, NJ, USA) at 77 K for 120 s. The fine crystalline hydrate/ice powder was immediately placed on a pre-cooled sample holder in a low-temperature chamber (153 K) and measured at ambient pressure with powder X-ray diffraction. PXRD patterns were obtained with the same diffractometer (PANanalytical Empyrean, Malvern Panalytical GmbH, Kassel, Germany) with CuKα radiation (λ = 0.15406 nm generated at 40 kV and 40 mA), automatic divergence, and antiscatter slits. The measurements took place in a continuous scan mode at a step width of 0.028 in the 2θ range of 8°–55° with a total scan time of 312 s. The diffracted X-rays were detected with the PIXcel^3D^ detector. The spectra were computationally processed to evaluate the hydrate structure using EVA v11.0.0.3 [49].

## 4. Conclusions

In this study, we investigated the effects of QMP sediments on the formation of mixed gas hydrates simulating natural reservoir conditions in QMP by means of Raman spectroscopy and X-ray diffraction. Through additional comparative experiments with quartz sand and without sediments, we were able to determine the specific influence of the QMP sediments on the hydrate formation.

By carrying out the formation experiments with/without sediments in batch reactors, time-resolved pressure information was recorded, which turned out to indicate the promoting effects of sediments on the hydrate nucleation and growth process. The promoting effects were enhanced by 32.7% in the group with the fine-grained QMP sediments compared to the system without sediments. This number was 23.5% for those in the group with quartz sand.

The Raman spectroscopic measurements revealed that the equilibrium curve of the mixed gas hydrates was not shifted with the presence of a limited amount of sediments. This is in agreement with the similar composition of the resulting hydrate phases in all series of tests at the end of the experiments. However, beyond the well-known heterogeneity of the hydrate phase, the development of the guest molecule enclathration varied in the three systems, resulting in differed initial hydrate compositions. The presence of sediments led to a lower initial concentration of CO_2_ in the hydrate phase. A coexisting solid phase in the system with QMP sediments was also observed, which depicted totally different Raman spectra compared to those from the typical structure II hydrate crystals. The existing hydrophobic minerals or organic compounds in QMP sediments might be a possible explanation, as they could have affected the arrangement of water molecules and inhibit the formation of crystalline hydrates. Instead of the growth of hydrate crystals, some specific points showed limited guest enclathration during the process.

These results can contribute to a better understanding of the complex mixed gas hydrate system in QMP. It also provides insights into structure II mixed gas hydrates in other natural hydrate reservoirs with a thermogenic gas source involving a series of guest species.

## Figures and Tables

**Table 1:** Results of gas-phase measurements before the formation of mixed gas hydrates in the high-pressure cell and the calculated hydrate composition from CSMGem.

No.	Sediments	P (MPa)	T (K)	Average mol%			
				CH_4_	C_2_H_6_	C_3_H_8_	CO_2_
Original gas				63	15	15	7
Test 1	/	2.2	274	47.41	19.06	19.08	14.45
Test 2	/	2.2	274	47.40	20.39	17.05	15.6
Test 3	QMP sediments	2.2	274	47.55	20.26	17.32	14.87
Test 4	QMP sediments	2.2	274	47.53	19.64	19.11	13.72
Test 5	Quartz sands	2.2	274	45.16	20.24	19.45	15.15
Test 6	Quartz sands	2.2	274	45.41	20.11	18.99	15.49
Average gas composition				46.74	19.95	18.55	14.81
Calculated composition of hydrate phase (CSMGem)				58.7	3.9	32.0	5.4

**Table 2:** Average composition of the hydrate phase at the beginning (Day 0) and at the end (Day 4) of the experiment under 3 different scenarios.

	Guest Component			
	CH_4_	C_2_H_6_	C_3_H_8_	CO_2_
Without sediment (Day 0)	31.6 ± 5.9	15.2 ± 5.1	26.0 ± 6.3	37.9 ± 7.3
Without sediment (Day 4)	43.6 ± 2.3	7.0 ± 0.5	38.6 ± 2.2	10.8 ± 1.2
Quartz sand (Day 0)	35.5 ± 4.4	18.6 ± 4.3	27.0 ± 4.5	18.9 ± 6.7
Quartz sand (Day 4)	43.7 ± 2.3	8.0 ± 1.4	38.7 ± 1.7	10.0 ± 1.6
QMP sediment (Day 0)	35.0 ± 7.5	21.1 ± 10.5	25.3 ± 8.4	18.5 ± 6.4
QMP sediment (Day 4)	47.1 ± 2.3	5.8 ± 1.0	36.4 ± 2.1	10.7 ± 2.0

**Table 3:** Summary of the Raman band assignments used in this study.

Component	Vibrational Mode	Cavity Type/Gas Phase	ν_measured_ (cm^−1^)	ν_literature_ (cm^−1^)	References
CH_4_	C–H stretching	Gas phase	2916	2916	[39]
		sII 5^12^	2912	2912	[2]
		sII 5^12^6^4^	2901	2901	
C_2_H_6_	C–C stretching	Gas phase	993	993	[2]
		sII 5^12^6^4^	991	992	
C_3_H_8_	C–C stretching	Gas phase	869	869	[39]
		sII 5^12^6^4^	877	877	
CO_2_	C–O stretching	Gas phase	1285	1285	[40]
		sII 5^12^6^4^	1274	1275	
	overtone bending	Gas phase	1387	1388	[40]
		sII 5^12^6^4^	1381	1381	

## Data Availability

Data are contained within the article.
